# A Joint LiDAR and Camera Calibration Algorithm Based on an Original 3D Calibration Plate

**DOI:** 10.3390/s25154558

**Published:** 2025-07-23

**Authors:** Ziyang Cui, Yi Wang, Xiaodong Chen, Huaiyu Cai

**Affiliations:** Key Laboratory of Opto-Electronics Information Technology of Ministry of Education, College of Precision Instrument and Opto-Electronics Engineering, Tianjin University, Tianjin 300072, China; 2023202037@tju.edu.cn (Z.C.); xdchen@tju.edu.cn (X.C.); hycai@tju.edu.cn (H.C.)

**Keywords:** calibration, camera and LiDAR, feature point, intelligent vehicle

## Abstract

An accurate extrinsic calibration between LiDAR and cameras is essential for effective sensor fusion, directly impacting the perception capabilities of autonomous driving systems. Although prior calibration approaches using planar and point features have yielded some success, they suffer from inherent limitations. Specifically, methods that rely on fitting planar contours using depth-discontinuous points are prone to systematic errors, which hinder the precise extraction of the 3D positions of feature points. This, in turn, compromises the accuracy and robustness of the calibration. To overcome these challenges, this paper introduces a novel 3D calibration plate incorporating the gradient depth, localization markers, and corner features. At the point cloud level, the gradient depth enables the accurate estimation of the 3D coordinates of feature points. At the image level, corner features and localization markers facilitate the rapid and precise acquisition of 2D pixel coordinates, with minimal interference from environmental noise. This method establishes a rigorous and systematic framework to enhance the accuracy of LiDAR–camera extrinsic calibrations. In a simulated environment, experimental results demonstrate that the proposed algorithm achieves a rotation error below 0.002 radians and a translation error below 0.005 m.

## 1. Introduction

In recent years, with the rapid development of autonomous driving [1] and intelligent robotics [2], multi-sensor fusion schemes have become the mainstream approach. In the domain of autonomous driving, cameras and LiDAR serve as core components of the perception system, each with distinct advantages and limitations. Cameras offer rich color and texture information, and image-based object detection and segmentation algorithms are relatively mature. However, their ability to estimate depth is limited, and their performance is highly sensitive to environmental conditions such as lighting [3]. In contrast, LiDAR provides accurate depth information and is robust to lighting variations, but the resulting point cloud data is typically sparse, which complicates object recognition and classification in complex environments [4]. Compared to single-sensor systems, multi-sensor systems deliver a richer and more accurate environmental perception. The prerequisite for effective fusion is the accurate determination of the extrinsic calibration between the LiDAR and the camera. However, precisely calibrating multiple sensors within a system remains a significant challenge.

Accurately extracting corresponding features from different data modalities is critical for successful LiDAR–camera extrinsic calibrations. Although numerous calibration methods have been proposed [5,6,7,8,9,10], obtaining consistent and reliable feature correspondences across images and point clouds remains a pressing issue. For example, while planar surfaces are relatively easy to extract from both modalities, they offer weak geometric constraints, thus limiting the calibration accuracy. Conversely, identifying corresponding points, such as checkerboard corners, in sparse point clouds is challenging. To address this, some methods [7,11,12,13] assume that depth-discontinuous points lie on the edges of planar surfaces and estimate feature locations by fitting these edges. However, due to the point cloud sparsity, these assumptions often introduce systematic errors, as depth-discontinuous points may not align precisely with actual edge locations. Meanwhile, 2D feature detectors based on image pixels, such as checkerboard corner detectors, can accurately identify targets in simple scenes but are susceptible to interference and misdetection in complex environments.

To address these limitations, this study proposes a novel 3D calibration plate integrating the depth gradient, localization markers, and corner point information, alongside an automatic LiDAR–camera calibration algorithm. At the image level, the localization markers on the calibration plate help the camera detect the plate position accurately, minimizing the interference from environmental factors. The corner features, centered on target points, allow the precise extraction of pixel coordinates. At the point cloud level, as the LiDAR scanning line sweeps through the gradient depth region of the plate, it generates scanning points. The intersection between a fitted line through these scanning points and the main plane of the calibration plate lies within the contour. By further fitting contour points from multiple LiDAR scan lines and computing their line intersections, the 3D coordinates of feature points can be accurately derived. Finally, using a feature point matching process, the transformation matrix between LiDAR and the camera is estimated.

The main contributions of this paper are as follows:

1. A novel 3D calibration plate is designed, integrating depth gradients, localization markers, and corner features;
2. A complete extrinsic calibration framework is developed, enabling an accurate feature point extraction across modalities;
3. A Spatial Clustering Algorithm for LiDAR based on the slope and distance (SCAL-SD) is proposed, tailored to the characteristics of LiDAR data.

## 2. Related Work

In the field of multi-sensor calibration, numerous innovative methods have been proposed, each contributing significantly to the advancement of calibration technologies [14,15,16]. In recent years, the demand for a higher accuracy and robustness has driven the development of many new approaches, particularly for LiDAR–camera calibrations. These methods can generally be classified into targetless and target-based calibration techniques.

Targetless calibration derives the relative transformation between the LiDAR and the camera by matching common environmental features captured by both sensors, such as edge lines, lane markings, and corner points [17,18,19,20]. Typically, the reflectance intensity of a LiDAR point cloud correlates positively with the grayscale values of corresponding image pixels. For instance, Pandey et al. [17] performed the calibration by maximizing mutual information between the LiDAR reflectance and image intensity. Bai et al. [18] extracted edge lines from both the image and point cloud data and optimized the extrinsic parameters using constraints derived from matched line features to achieve pixel-level fusion. However, this method requires a manual annotation to establish the correspondence between edge features across modalities, increasing the calibration workload. Similarly, Ma et al. [19] utilized lane lines, which are usually highly reflective and visually distinct from the surrounding environment, to establish correspondences for calibration. Nonetheless, this method is only applicable in scenes where lane markings are clearly visible. Moreover, the accuracy of these methods is highly dependent on the quality of the input images and point clouds.

With the rapid development of deep learning, increasing attention has been directed toward its application in sensor calibration tasks [6,21,22,23]. Iyer et al. [6] proposed CalibNet, which integrates traditional calibration steps into a convolutional neural network, trained by maximizing both the geometric and photometric consistency between LiDAR and image data. Feng et al. [21] employed SIFT [24] and the ISS [25] to extract keypoints from images and point clouds, respectively. These keypoints and their surrounding regions were then input into CNNs [26] and PointNet [27] to learn corresponding 2D–3D features. Lv et al. [22] introduced LCCNet, an end-to-end deep learning framework capable of real-time predictions of extrinsic calibrations. The use of neural networks can simplify the calibration process and enhance accuracy. However, many deep learning-based methods still rely heavily on target-based calibration for training and ground truth verification [28].

Target-based calibration methods utilize specific calibration targets to assist in LiDAR–camera registration and can be further divided into vector constraint and feature point methods. The vector constraint method was first introduced by Zhang [8], who used a checkerboard plane to detect image corners and fit them to a plane, aligning the corresponding 3D planes from LiDAR using geometric constraints. Unnikrishnan et al. [29] obtained extrinsic parameters by minimizing the distance between planes in the LiDAR and camera coordinate frames. Geiger et al. [30] enhanced efficiency by placing multiple calibration plates in the scene and completing the calibration using constraints between planes in a single frame. However, vector constraint methods often suffer from a high computational cost and operational complexity.

The feature point method estimates the transformation matrix between sensors by identifying the same target’s position in different data modalities and applying point-to-point geometric constraints. Compared to other constraint types, point-to-point constraints offer more independent equations for optimization [7]. The effectiveness of feature point methods [5,11,12,13] largely depends on the accurate extraction of corresponding point features from point clouds and image data, which is closely tied to the design of the calibration board.

**Figure 1:**
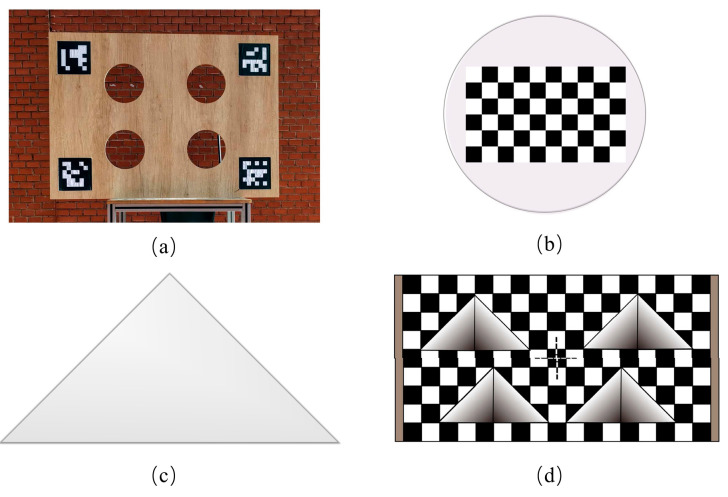
Various calibration plate patterns. (**a**) A rectangular plate featuring void edge contours and ArUco marker information. (**b**) A circular plate incorporating a checkerboard pattern. (**c**) A triangular plate containing planar edge features. (**d**) A multidimensional calibration plate combining localized depth gradients and checkerboard information.

As illustrated in Figure 1a, Beltrán et al. [11] extracted the spatial coordinates of circular hole centers by fitting contours using depth discontinuities generated from LiDAR scans of a flat calibration plate. Simultaneously, the QR code on the plate enabled them to obtain the 3D coordinates of feature points in the camera coordinate system. In Figure 1b, Liu et al. [12] designed a circular calibration plate containing a checkerboard pattern, where the checkerboard center coincided with the center of the circular plane. The 3D coordinates were estimated by fitting the circular contour from edge points, while the checkerboard aided in obtaining the corresponding 2D pixel coordinates. Figure 1c shows Park et al. [13] using a triangular calibration plate. They fitted straight lines along the plate’s edges using depth discontinuity points and identified feature points at the intersection of these lines. All the above methods rely on fitting planar contours from depth discontinuity points. However, this introduces systematic errors, as these points may not perfectly align with the actual contour. In Figure 1d, Cai et al. [5] introduced a calibration plate with a depth gradient. By fitting lines to scanning points within the gradient region and computing their intersections with the plate’s main plane, they could accurately determine contour points (i.e., feature points), effectively reducing the systematic error. However, the position of the feature point is influenced by the intersection between the LiDAR scan line and the hollow contour of the calibration plate, rather than being a fixed point on the calibration plate. Consequently, the pixel coordinates of the feature points cannot be determined solely from image data, making it difficult to ensure their accuracy.

Given these challenges, the accurate extraction of matching feature points from both point clouds and images has become a critical step in achieving a precise extrinsic calibration. To address this issue, this paper proposes a novel joint calibration method for LiDAR–camera systems. The proposed method incorporates depth gradients, localization icons, and corner point features to extract corresponding points from multimodal data, which are then used to constrain the extrinsic calibration matrix. This approach not only overcomes the challenge of extracting features from sparse point clouds but also improves the calibration accuracy, offering a simple yet effective solution for LiDAR–camera joint calibrations.

## 3. Methodology

The characteristics of the calibration plate play a critical role in enabling the accurate extraction of corresponding features from point clouds and images. Given that cameras are sensitive to semantic information and LiDAR is sensitive to depth information, this paper presents a novel 3D calibration plate that integrates depth gradient, localization icons, and corner point features.

**Figure 2:**
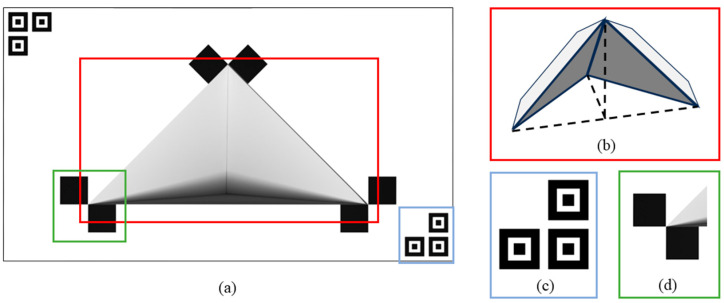
The original 3D calibration plate. (**a**) The front side of the calibration plate. (**b**) The depth gradient region of the calibration plate. (**c**) Positioning icons on the calibration plate. (**d**) The corner point information centered on the vertex of the triangle cavity.

As shown in Figure 2a, an isosceles right triangular cavity is cut into the center of the calibration plate, with a custom hollow tetrahedral baffle fixed behind it. As shown in Figure 2b, the base of the baffle is an isosceles right triangle, with dimensions that match those of the cavity on the calibration plate. Once assembled, the surface of the calibration plate appears seamless when viewed from the front, creating a localized depth gradient region. Furthermore, assigning corner point information centered at each vertex of the cavity facilitates the extraction of pixel coordinates of the feature points from the image. Additionally, placing a positioning marker at the diagonal of the calibration plate helps the camera identify the region of interest (ROI) for corner detection, effectively reducing interference from environmental factors.

Based on this design, we propose a novel LiDAR–camera joint automatic calibration algorithm. The algorithm extracts matched point features from point cloud and image data, optimizes the objective function using geometric constraints, and outputs the transformation matrix. Where the objective function and the transformation matrix H are defined as follows:

(1) $$\left(H*\right)=arg\underset{\left\{h_{\mathrm{mn}}\right\}}{\min}\sum_{{\tilde{x}}_{i}^{2D},{\;\tilde{x}}_{i}^{3D}}\left\|s_{i}\cdot {\tilde{x}}_{i}^{2D}-H\cdot {\tilde{x}}_{i}^{3D}\right\|,$$

(2) $$H=\left(\begin{array}{ccc} f_{x} & 0 & c_{x}\\ 0 & f_{y} & c_{y}\\ 0 & 0 & 1 \end{array}\right)*\left(R|t\right)=\left(\begin{array}{ccc} h_{11} & h_{12} & \begin{array}{cc} h_{13} & h_{14} \end{array}\\ h_{21} & h_{22} & \begin{array}{cc} h_{23} & h_{24} \end{array}\\ h_{31} & h_{32} & \begin{array}{cc} h_{33} & h_{34} \end{array} \end{array}\right),$$

Here, $s_{i}$ is the scale factor of the ith projected feature point; H denotes the transformation matrix; ${\tilde{x}}_{i}^{2D}$ represents the homogeneous coordinates of feature point in the pixel coordinate system; and ${\tilde{x}}_{i}^{3D}$ represents the homogeneous coordinates of feature point in the LiDAR coordinate system. R and t denote the rotation matrix and translation vector from LiDAR to the camera.

**Figure 3:**
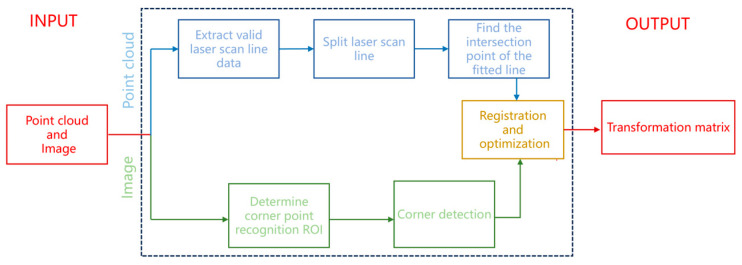
Calibration flowchart.

This method does not impose strict requirements on the relative positions of the sensors but adheres to two reasonable conditions: (1) the calibration plate must be visible in the field of view of both the LiDAR and the camera; and (2) the hollow contours on the calibration plate must be clearly detectable by both sensors. Specifically for LiDAR, at least two scan lines should pass through the cavity region. As illustrated in Figure 3, the proposed calibration process proceeds as follows:

1. At the point cloud level, extract the 3D coordinates of the triangular cavity vertices of the calibration plate from the point cloud.
2. At the image level, identify the corresponding 2D coordinates from the image.
3. Perform 2D–3D feature matching and alignment.

### 3.1. Extraction of 3D Coordinates from Point Clouds

Due to the sparsity of LiDAR data, the scanned points rarely coincide precisely with the vertices of the triangular cavity. Therefore, a fitting process is required: lines are fitted to the contour points of the triangular cavity, and the intersections of these lines determine the vertex coordinates. Some existing methods approximate contour points using depth discontinuity points, which introduces systematic errors. In contrast, this study incorporates a depth gradient plane behind the cavity. By fitting straight lines to points on both the main plane and the depth gradient plane, their intersection yields accurate contour points.

The procedure for extracting the 3D coordinates of the triangular cavity vertices consists of three steps:

1. Apply the SCAL-SD algorithm to cluster the point cloud.
2. Filter and extract relevant line segments to identify contour points.
3. Use known cavity dimensions to compute the 3D coordinates of the cavity vertices.

The first step involves applying the SCAL-SD algorithm to cluster the point cloud data. Initially, pass-through filters are applied in the three cartesian coordinates to remove points outside the area where the target is to be placed. Subsequently, SCAL-SD is used to perform clustering on the filtered data. Unlike DBSCAN [31], SCAL-SD segments the point cloud into multiple sub-clouds based on scan line planes, utilizing the inherent working principle of LiDAR. These sub-clouds are further clustered using both distance and slope attributes. Each resulting cluster is considered a line segment composed of scan points, referred to as a line cloud. The implementation details of the SCAL-SD algorithm are described below.

**Figure 4:**
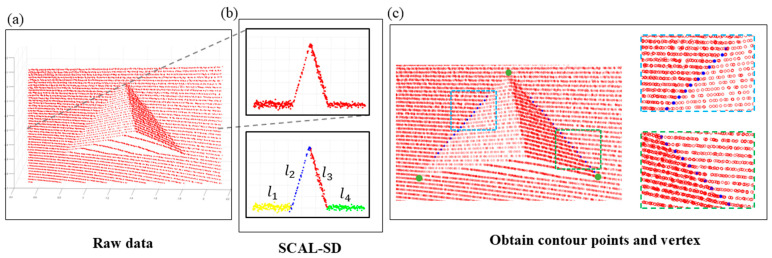
(**a**) All scan points located on the calibration plate. (**b**) The upper panel shows the scan points generated by a specific LiDAR scan line in (**a**); the lower panel illustrates the division of these scan points into four line segments, denoted as $\left\{l_{i}\right\}$, using the SCAL-SD method. The intersection of $l_{1}$ and $l_{2}$ lies on the left contour of the cavity, while the intersection of $l_{3}$ and $l_{4}$ lies on the right contour. (**c**) Blue points indicate the hollow contour, and green points denote the hollow vertices.

**Cluster point clouds based on slope and distance.** As shown in the top panel of Figure 4b, let φᵢ denote the point cloud generated by the ith LiDAR scan line. The scan points within φᵢ are sorted according to their yaw angles. The first three sorted points, denoted as $Q_{0}$, $Q_{1}$, and $Q_{2}$, are evaluated using Equation (3) to determine whether they belong to the same class:

(3) $$\left\{\begin{array}{c} \frac{\left\|\vec{Q_{0}Q_{1}}\times \vec{Q_{1}Q_{2}}\right\|}{\left\|\vec{Q_{0}Q_{1}}\right\|}<D_{thre}\\ \begin{array}{c} \left\|\vec{Q_{0}Q_{1}}\right\|<D_{thre}\\ \left\|\vec{Q_{1}Q_{2}}\right\|<D_{thre} \end{array} \end{array}\right.,$$

Here, $D_{thre}$ represents the maximum allowable offset distance. If the conditions in Equation (3) are satisfied, the points are stored in the candidate line segment l; otherwise, they are discarded, and the evaluation continues with the next points. Once the candidate line segment $l=\left\{Q_{j},Q_{j+1},\ldots ,Q_{k}\right\}$ is formed, the inclusion of the next point $Q_{k+1}$ is determined using Equation (4):

(4) $$\left\{\begin{array}{c} \left\|\vec{Q^{\prime }Q_{k+1}}\times \vec{v}\right\|<D_{thre}\\ \left\|\vec{Q_{k}Q_{k+1}}\right\|<D_{thre} \end{array}\right.,$$

In this equation, $Q^{\prime }$ and $\vec{v}$ represent the centroid and the direction vector of the current candidate segment l, respectively. If the conditions are satisfied, $Q_{k+1}$ is added to l, and both the centroid and direction vector are updated. The process then proceeds with the next point in sequence.

**Figure 5:**
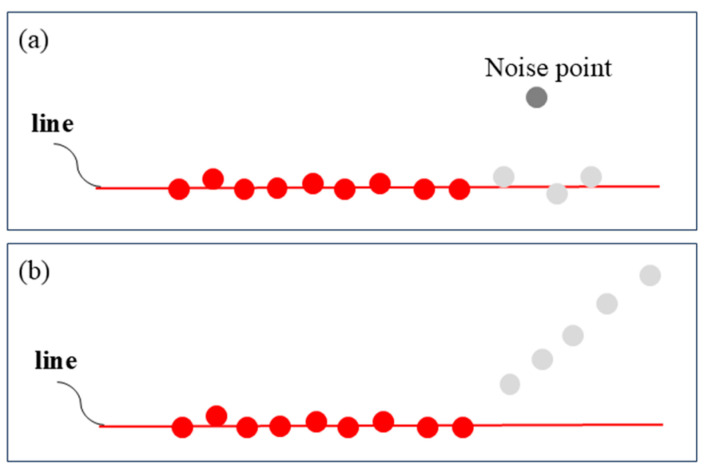
Red points represent the points that have been classified to the line, gray points represent the points to be measured, and black points represent the perturbation points. (**a**) Although the noise point deviates from the line, the algorithm still classifies the gray point to the line because the subsequent points are located near the line. (**b**) The clustering to the line stops when several subsequent points from the first gray point deviate from the line.

**Resist interference caused by noise points.** During the clustering process, noise may affect the results. As illustrated in Figure 5a, if a perturbation point deviates from the straight line along which the candidate line segment l lies, but the subsequent points remain aligned with the line, the algorithm skips the perturbation point and continues the evaluation. In contrast, as shown in Figure 5b, if both the current point and its subsequent points deviate from the line, the clustering process for segment l is terminated. Finally, whether l is categorized as part of the line cloud $\left\{l_{i}\right\}$ is determined by checking whether the number of points in l exceeds a predefined threshold. After SCAL-SD processing, the scanned points from a single LiDAR scanning plane are divided into distinct line segments, as shown in the bottom panel of Figure 4b.

In the second step, the target line segments are filtered, and the 3D coordinates of the contour points are calculated. In the previous step, the SCAL-SD algorithm segmented the LiDAR scan points from each scan line into multiple line segments, collectively forming the line cloud $\left\{l_{i}\right\}$. As illustrated in Figure 4b, after SCAL-SD processing, the point cloud in the depth gradient region of the calibration plate is divided into four line segments, labeled $l_{1},\;l_{2},l_{3},\;\mathrm{and}\;l_{4}$ from left to right. Based on the geometric structure of the calibration plate, the target group $\left\{l_{1},l_{2},l_{3},l_{4}\right\}$ is selected from $\left\{l_{i}\right\}$. Each line segment is fitted using the least squares method to obtain its linear expression. Compute the intersections of $l_{1}$ and $l_{2}$ and $l_{3}$ and $l_{4}$, which lie on the left and right contours of the triangular cavity, respectively.

In the third step, the 3D coordinates of the cavity vertices are determined using the known dimensions of the cavity. As shown in Figure 4c, each scan line that passes through the cavity generates one contour point on each of the two lateral edges of the triangle. These contour points are grouped and denoted as $\left\{P_{iL}\right\}$ and $\left\{P_{iR}\right\}$, respectively. Linear expressions for the lateral edges are obtained by fitting the corresponding contour points, and the intersection of these two lines is the vertex $v_{c}$ at the top of the triangular cavity.

Given the known length t of the cavity’s side edges, the 3D coordinates of the other two triangle vertices can be computed using Equation (5):

(5) $$\left\{\begin{array}{c} v_{L}=v_{C}-t*\vec{S_{L}}\\ v_{R}=v_{C}-t*\vec{S_{R}} \end{array}\right.,$$

Here, $\vec{S_{L}}$ and $\vec{S_{R}}$ represent the normalized direction vectors of the lines corresponding to the two lateral edges of the cavity. The complete process for extracting the 3D coordinates of the triangular cavity vertices from the point cloud is summarized in Algorithm 1.

**Algorithm 1:** Algorithm for Feature Points Extraction from Point Cloud using SCAL-SD
Input: original 3D point cloud
Output: 3D Coordinates of Feature Points $\left\{v_{C},v_{L},v_{R}\right\}$
1.     Group the original 3D point cloud into subsets based on pitch angle: $\left\{\varphi \right\}$2.     For each $\varphi_{i}\epsilon \left\{\varphi \right\}$ do3.               Apply the SCAL-SD to the points in $\varphi_{i}$ to generate line clouds: $\left\{l_{i}\right\}$4.               Filter the line clouds $\left\{l_{i}\right\}$ to obtain the target line segments $\left\{l_{1},l_{2},l_{3},l_{4}\right\}$, and derive the contour points: $P_{iL}$$,\;P_{iR}$5.     End for6.     Fit lines to the contour points $\left\{P_{iL}\right\}$$,\;\left\{P_{iR}\right\}$, and determine their intersection point: $v_{C}$7.     Based on prior knowledge, determine the positions of the remaining two vertices: $v_{R}$$,\;v_{L}$


### 3.2. Capture 2D Coordinates of Feature Points

On the calibration plate, the hollow vertices align with the corner positions, allowing the pixel coordinates of the feature points to be obtained through corner point detection. In practical calibration scenarios, the choice of corner extraction technique depends on the complexity of the background. When the background is relatively simple or exhibits clear contrast with the calibration board, traditional corner detection methods, such as the Harris detector, can effectively identify corners on the board. However, in complex environments containing abundant semantic information, background interference can hinder accurate detection of target corners, making it difficult to determine the pixel coordinates of the feature points.

**Figure 6:**
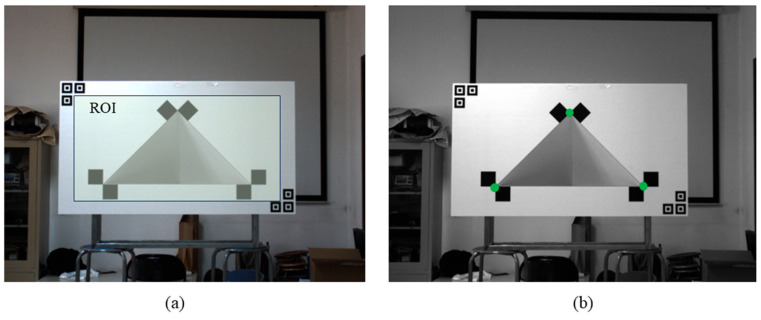
Capturing feature points in 2D coordinates under the pixel coordinate system. (**a**) The region of interest (ROI) for the corner identification is determined by detecting the localization icon. (**b**) Within the ROI, the three pixels with the highest corner response values are selected as feature points.

To address this issue, a localization icon is added to the edge of the calibration plate. As illustrated in Figure 6, the camera uses these icons to identify the region of interest (ROI) for corner detection. This strategy not only mitigates the impact of environmental interference but also enhances the efficiency of subsequent corner recognition. Corner detection within the ROI is performed using the ChESS algorithm [32], which outputs a quantitative measure indicating the likelihood of a candidate point being a true corner. Following non-maximum suppression, the three points with the highest likelihood scores are selected as the 2D pixel coordinates of the feature points. The final recognition results are presented in Figure 6b.

### 3.3. 2D–3D Feature Point Matching and Alignment

After establishing correspondences between 2D and 3D feature points using relative positional information, the 3D coordinates $\left\{\left(x_{1},y_{1},z_{1}\right),\left(x_{2},y_{2},z_{2}\right),\ldots ,\left(x_{n},y_{n},z_{1n}\right)\right\}$ and the corresponding 2D pixel coordinates $\left\{\left(u_{1},v_{1}\right),\left(u_{2},v_{2}\right),\ldots ,\left(u_{n},v_{n}\right)\right\}$ are obtained, where $\left(x_{i},y_{i},z_{i}\right)$ and $\left(u_{i},v_{i}\right)$ represent the 3D and 2D coordinates of a vertex of the triangular cavity, respectively. These 2D–3D point pairs are substituted into Equation (1) to estimate the transformation matrix. The initial estimate of H is obtained via the PnP algorithm [33], followed by nonlinear optimization using the Levenberg–Marquardt algorithm to yield the optimal alignment matrix.

## 4. Simulation and Experiment

In this study, the proposed calibration method is evaluated through both simulations and real-world experiments. First, the algorithm’s performance is quantitatively assessed on the Gazebo 11 simulation platform. Subsequently, the joint calibration of the LiDAR and camera is conducted in a real-world environment, and the resulting calibration accuracy is evaluated. To investigate the impact of the number of calibration plate poses and the presence of noise on the calibration performance, multiple sets of experiments are conducted. The results are then compared to those obtained from several mainstream calibration methods.

During the experimental procedure, allowing as many laser scan lines as possible to cover the depth gradient region significantly improves the accuracy of the 3D feature point localization. Based on empirical observations, ensuring at least six valid scan lines is sufficient to maintain the algorithm’s performance. For most commercially available LiDAR models, meeting this requirement is generally feasible. In our implementation, the RANSAC-PnP function from the OpenCV library was used to estimate the initial transformation matrix. Compared to the standard PnP algorithm, RANSAC-PnP offers a greater robustness against outliers.

### 4.1. Simulation Experiments

In real-world settings, it is often challenging to obtain the ground truth of the relative pose between the camera and LiDAR. Therefore, a simulation experiment is conducted on the Gazebo platform, which enables access to the true relative position between the sensors. Furthermore, the intrinsic parameters of the sensors can be precisely defined in the simulation, ensuring that the evaluation depends solely on the accuracy of the joint calibration algorithm.

Following the approach in [11], the translational error $e_{t}$ and rotational error $e_{r}$ are employed as metrics to evaluate the calibration performance:

(6) $$e_{t}=\left\|\hat{t}-t\right\|,$$

(7) $$e_{r}=∠\left({\hat{R}}^{-1}R\right),$$

Here, $\hat{t}$ and $\hat{R}$ denote the translation vector and rotation matrix estimated through the calibration, while t and R represent the ground truth translation vector and rotation matrix, respectively.

#### 4.1.1. The Influence of the Number of Calibration Plate Positions

During the calibration process, the number of calibration plate positions directly affects the number of generated feature points. A higher number of feature points provides stronger constraints for estimating the alignment matrix. To investigate the impact of the calibration plate positioning and determine the optimal number of positions for an accurate calibration, a series of experiments were conducted. To better simulate real-world conditions, Gaussian noise was added to the camera intensity and LiDAR data. During the experiments, the calibration plate was moved within the overlapping field of view of the sensors, and 50 independent trials were performed for each configuration.

**Figure 7:**
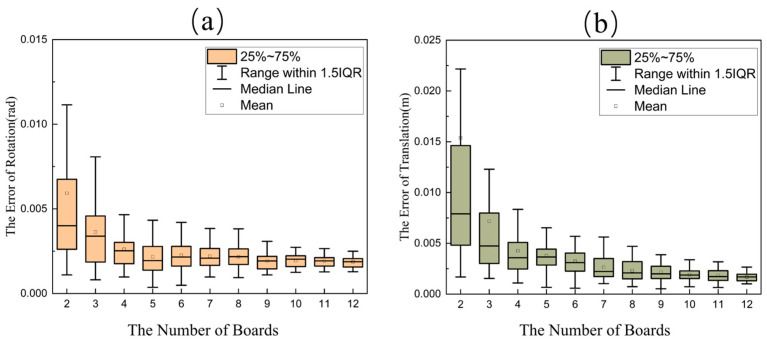
The effect of the number of calibration plate positions on the calibration results. (**a**) The effect of the number of calibration plates on the rotation error. (**b**) The effect of the number of calibration plates on the translation error.

The final results are presented in Figure 7. In the box-and-whisker plot, the boxes represent the interquartile range (25th to 75th percentiles), the solid line indicates the median, and the small square denotes the mean. Experimental findings demonstrate that the proposed method requires as few as two frames of matched data to achieve the joint calibration between the LiDAR and camera. As the number of calibration plate poses increases, both rotational and translational errors exhibit a decreasing trend. This decline continues up to 10 frames, beyond which adding more data does not significantly reduce the calibration error. Ultimately, the rotational error stabilizes at approximately 0.0018 radians, and the translational error converges to around 0.0015 m.

By analyzing the impact of the number of calibration plate positions, unnecessary sensor measurements can be effectively avoided, enabling accurate and reliable calibration results.

#### 4.1.2. Impact of Noise

To further validate the calibration accuracy and robustness of the proposed algorithm, and to investigate the impact of varying noise levels, a series of comprehensive experiments were conducted on the Gazebo simulation platform. During these experiments, different levels of Gaussian noise were added to the LiDAR point cloud, while fixed Gaussian noise with a standard deviation of σ=0.007 was applied to the camera images. The calibration methods proposed by Zhou et al. [23], Park et al. [13], and the method proposed in this study were each used to calibrate the LiDAR–camera system. For each noise level, 50 independent trials were conducted per method, and the calibration results were recorded. The detailed results are presented in Table 1.

The experimental results indicate that the calibration errors of all methods tend to increase as the noise intensity rises, suggesting that joint calibration algorithms are sensitive to point cloud noise. Specifically, as the noise intensity increases, the rotation error of Zhou et al.’s algorithm [23] grows from 0.0032 rad to 0.0045 rad, while the translation error increases from 0.0084 m to 0.0210 m. In contrast, the calibration errors of both the proposed method and the algorithm by Park et al. [13] remain relatively low across varying noise levels, with smaller increases in error. Notably, the proposed method exhibits the smallest increase in the calibration error among all methods and is the only one to maintain translation errors below 0.01 m. These comparative results demonstrate the robustness and reliability of the proposed method in mitigating noise disturbances and confirm its effectiveness and accuracy in achieving the joint calibration between the camera and LiDAR under different environmental conditions.

### 4.2. Experiments Based on Real Scenarios

Simulation experiments are conducted to systematically and accurately evaluate the performance of the proposed algorithm. To further verify its practical applicability, real-world experiments are performed to assess whether the method meets the requirements of intended applications.

**Figure 8:**
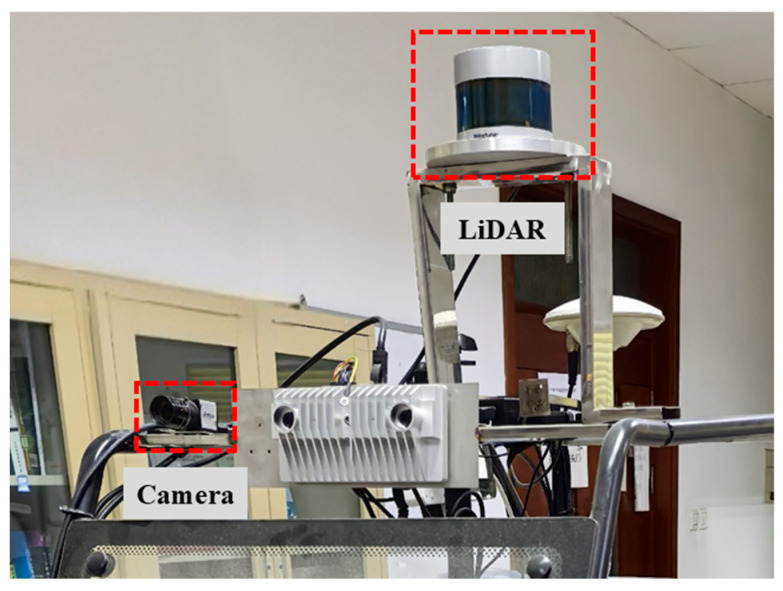
Sensors used in real-world experiments and their relative configuration.

As illustrated in Figure 8, the LiDAR and camera are mounted on the frame of the experimental vehicle. The LiDAR model used is the Velodyne VLP-32C, which features a detection range of 0–200 m, a vertical field of view from −25° to 15°, a horizontal field of view of 360°, and both horizontal and vertical resolutions of 0.4°. The minimum vertical angular resolution is 0.33°, with a detection accuracy of ±5 cm for distances under 50 m and ±10 cm for distances between 50 and 200 m. The camera model is the A5131CU210 from Archforce Technology, with a resolution of 1280 × 1024 pixels and a pixel size of 4.8 μm × 4.8 μm. The lens model, MH0820S (also from Archforce Technology), offers a field of view of 60.8° × 42.7°, a focal length of 8 mm, and a lens distortion of less than 0.1%.

**Figure 9:**
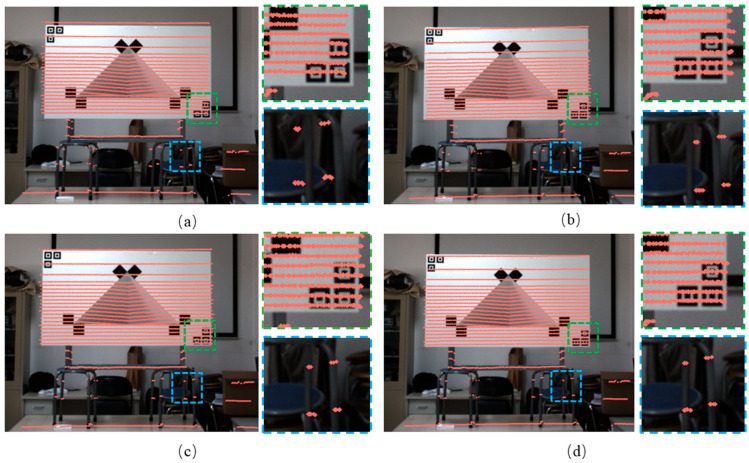
Reprojection results. (**a**) Reprojection results using Zhou’s algorithm. (**b**) Reprojection results using Park’s algorithm. (**c**) Reprojection results using Cai’s algorithm. (**d**) Reprojection results using the proposed method.

The LiDAR and camera are calibrated using the method proposed in this paper, and the point cloud data are reprojected onto the image based on the resulting calibration parameters. The reprojection outcomes are compared with those obtained using the methods of Zhou et al. [23] and Park et al. [13], as shown in Figure 9. The reprojection results obtained using the methods of Zhou and Park exhibit significant deviations, while those based on Cai’s method also display noticeable shifts. In contrast, the reprojected point cloud closely aligns with the corresponding pixel targets when calibrated using the method proposed in this paper. These experimental results demonstrate that fitting straight lines to the scan points located in the depth gradient region of the calibration plate, and computing their intersection with the main plane of the plate to determine contour points, can effectively reduce the systematic error and achieve the accurate estimation of the extrinsic parameters between the LiDAR and the camera.

To quantitatively assess the proposed calibration method, the reprojection error is used as the evaluation metric and compared against other feature point calibration approaches, as shown in Table 2. Among all listed methods, the proposed approach achieves the lowest reprojection error. These results further validate that accurate 3D coordinates of feature points can be obtained by determining contour points through line fitting and the intersection analysis, ultimately enabling the precise calibration between the LiDAR and camera systems.

## 5. Conclusions

In this paper, we propose a novel extrinsic calibration method for LiDAR and camera systems. The method extracts hollow contour points by fitting a straight line to scan points within the depth gradient region of a specially designed calibration plate and determining their intersection with the plate’s main plane. Subsequently, a straight line is fitted to these contour points to accurately estimate the 3D positions of feature points. This approach enables the precise extraction of the 3D feature information from sparse point clouds, effectively mitigates systematic errors, and significantly enhances the accuracy and robustness of the calibration.

Additionally, by combining localization icons with corner information, the method achieves the accurate extraction of the pixel coordinates of feature points from images while minimizing the interference from surrounding environments. A rigorous and systematic calibration framework is constructed to improve the precision of the extrinsic calibration between the LiDAR and camera systems.

The calibration technique introduced in this work is both innovative and robust, offering a significant improvement in sensor calibration accuracy. Compared to the LiDAR used in our experiment, newer LiDAR systems with more laser channels and a higher scanning accuracy have already been developed. According to the underlying calibration principles, these systems are expected to yield even more accurate results. Future research will explore the extension of this method to other types of LiDAR and camera systems. Furthermore, as deep learning holds promise for simplifying the calibration process and enhancing accuracy, future efforts will focus on the development of neural network-based joint calibration algorithms for LiDAR–camera systems.

## Figures and Tables

**Table 1:** Effects of different noise intensities on calibration methods.

Gaussian Noise (m)	Zhou et al. [23]	Park et al. [13]	Ours
0.005	0.0032 rad/0.0084 m	0.0026 rad/0.0059 m	**0.0018 rad/0.0010 m**
0.010	0.0040 rad/0.0139 m	0.0029 rad/0.0078 m	**0.0018 rad/0.0029 m**
0.015	0.0045 rad/0.0210 m	0.0030 rad/0.0103 m	**0.0020 rad/0.0055 m**

**Table 2:** Reprojection errors of different methods.

Calibration Method	Reprojection Errors/(Pixel)
Cai et al. [5]	1.8312
Xie et al. [7]	2.9700
Park et al. [23]	2.7345
Zhou et al. [13]	3.2162
Ours	**1.1957**

## Data Availability

Data are contained within the article.
